# Molecular treatments to reduce catabolic effects in human meniscus explant models

**DOI:** 10.1016/j.ocarto.2025.100618

**Published:** 2025-04-30

**Authors:** Amanda Sjögren, Karin Lindblom, Aleksandra Turkiewicz, Martin Englund, Patrik Önnerfjord

**Affiliations:** a Lund University, Faculty of Medicine, Department of Clinical Sciences Lund, Orthopaedics, Clinical Epidemiology Unit, Lund, Sweden; b Lund University, Faculty of Medicine, Department of Clinical Sciences Lund, Section for Rheumatology and Molecular Skeletal Biology, Lund, Sweden

**Keywords:** Meniscus, Explant model, Human tissue, Proteomics, Osteoarthritis

## Abstract

**Objectives:**

1. To validate catabolic meniscus explant models induced by cytokines: interleukin-6 ​+ ​interleukin-6 receptor ​+ ​tumor necrosis factor alpha (IL6/TNF) and oncostatin M ​+ ​tumor necrosis factor alpha (OSM/TNF). 2. To evaluate three potential anti-catabolic treatments: i) dexamethasone (DEX), ii) a Link-N peptide (Link-N) and iii) a peptide from chondroadherin (CKF).

**Design:**

Healthy lateral menisci from deceased donors (n ​= ​6; age ​= ​25–70 years, 4 males, 2 females), were sliced and randomized for experimental groups (combinations of the catabolic models and anti-catabolic treatments) and a control group. Culture media were analyzed, every third day until day 18, by mass spectrometry-based proteomics. Linear mixed effect models were used to estimate differences in protein abundances between groups.

**Results:**

A total of 662 proteins were identified in all menisci. Cytokine-treated meniscus explant models showed increased release of osteoarthritis-related proteins such as matrix metalloproteinases (MMPs). For example, MMP1: IL6/TNF vs. ctrl; log2 fold-change 2.2 95 ​% confidence interval [1.8, 2.5] and OSM/TNF vs. ctrl; log2 fold-change 2.8 [2.4, 3.1]. There was no treatment effect in explant meniscus with the addition of either Link-N or CKF. Treatment effects were, however, evident with the addition of DEX. For example, MMP1: IL6/TNF ​+ ​DEX vs. ctrl; log2 fold-change −1.8 [-2.2, −1.4] and OSM/TNF ​+ ​DEX vs. ctrl; log2 fold-change −0.3 [-0.7, 0.04].

**Conclusion:**

We confirmed that both catabolic models induce changes in osteoarthritis-related proteins. DEX treatment is effective in mitigating the catabolic response in meniscus explant models and may be further explored for its effects in the treatment of meniscus degeneration.

## 1 Introduction

One key tissue of the knee is the meniscus, which contributes to joint stability and distributes loads via the complex arrangement of its extracellular matrix (ECM) [1]. The meniscus has been reported to often be affected already at an early stage of knee osteoarthritis (OA) development [2] and injury to the meniscus increases the risk of developing symptomatic knee OA [3]. Therefore, further studies of the meniscus have potential to contribute to the development of new therapeutics targeting OA. One way to study human disease events is to mimic the native environment by explant models, with the advantage of having enhanced control when studying the tissue *ex vivo* [4,5]. Human meniscus explant models have previously been reported in regard to IL-1β treatment [6], injury [7] and injury repair [[8], [9], [10]]. The addition of cytokines to *ex vivo* tissue cultures could mimic inflammation processes, which is believed to play a role in OA pathophysiology [11]. Thus, in the current study, we built upon our previously established catabolic explant disease models using cytokine treatments of human meniscus [12]. Here, however, we extend the two models with addition of three molecules with the potential to reduce the catabolic processes in the tissue: 1) dexamethasone, a hormone with anti-inflammatory and immunosuppressive properties [13], hereafter referred to as DEX; 2) a Link-N peptide (DHLSDNYT; aa 1–8), which has previously been suggested to act as an inhibitor of catabolic processes in bovine intervertebral disc [14], hereafter referred to as Link-N. Further, a longer variant (aa 1–16) of the peptide had an anti-catabolic effect in osteoarthritic disease explant models in human cartilage [15]; and 3) a C-terminal heparin binding peptide from chondroadherin (CKFPTKRSKKAGRH), which binds to cell-surface proteoglycans [16] and modulates cell behavior, hereafter referred to as CKF. Our main aims were 1) to validate our previously published catabolic models in an independent setting and 2) to evaluate the three potential treatments by studying proteomic response in the explant culture media.

## 2 Method

### 2.1 *Ex vivo* tissue culture preparation

**Fig. 1:**
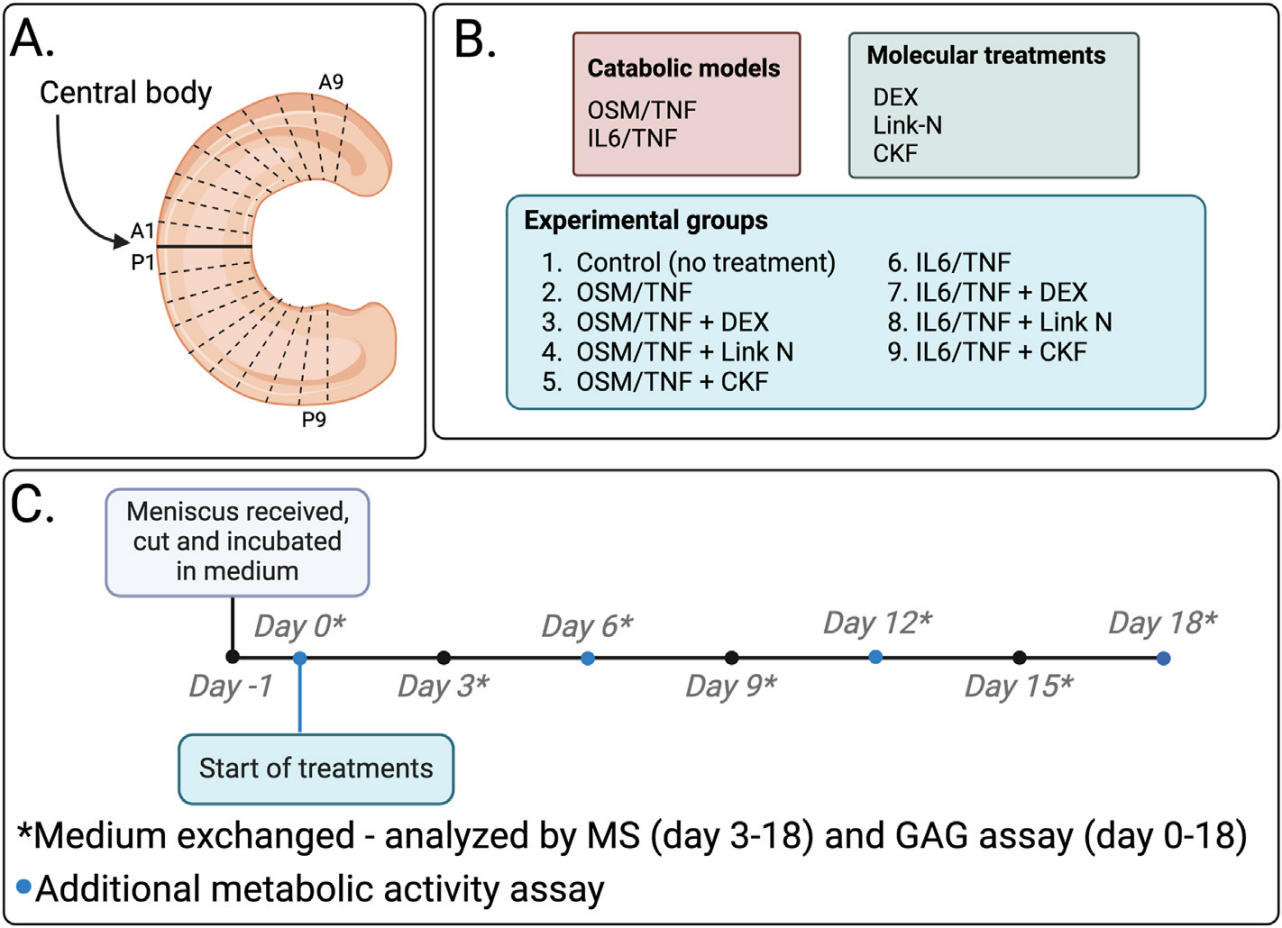
Project study design. **A.** Each lateral meniscus was divided into 18 slices. Nine slices below (posterior; P1–P9) and nine slices above (anterior; A1-A9) the central body. **B.** We used two previously established catabolic disease models: OSM/TNF (oncostatin-M and tumor necrosis factor alpha) and IL6/TNF (interleukin-6 and tumor necrosis factor alpha) [13]. In addition, we used molecules with the potential to inhibit the catabolic processes: DEX (dexamethasone), a Link-N peptide (from Link protein) and a CKF peptide (from chondroadherin). This resulted in a total of 9 different experimental groups for each meniscus, including control without treatment. **C.** Slices from the meniscus were left for one day to equilibrate in culture medium before start of treatments at day 0. Then, 80 ​% of the medium was changed every third day; it was saved for mass spectrometry (MS) analysis (day 3–18) and analysis of glycosaminoglycan (GAG) release (day 0–18). Metabolic activity by alamarBlue at day 0, 6, 12 and 18 (blue dots) was performed to ensure that cells were viable.

Lateral menisci from six deceased human donors without known joint disease (age ​= ​25–70 years, 4 males and 2 females) were extracted within 48 ​h post-mortem. The experiment for each of the biological samples will be referred to as MEX5.1-MEX5.6. For a meniscus to be eligible for the study, it needed to have a normal intact appearance, based on the macroscopic Pauli grading system [17] and we excluded donors with an age above 75. Synovial and fat tissue was removed and then, each meniscus was divided by the central body into an anterior and posterior part. Nine slices were obtained from both the anterior (A1-A9) and the posterior (P1–P9; Fig. 1A) half. The wet weight was noted (Supplementary table 1) and used for adjustment in the quantitative analysis. Meniscal experiments were conducted at different times to ensure tissue viability immediately after extraction. The sample size was chosen considering the number of available samples and the time constraints of the experimental procedure. The sample collection and analysis have been approved by the regional ethical review committee.

### 2.2 Tissue culture and treatments

The culture process and assay analyses have previously been described in Ref. [12]. In short, each meniscal slice was incubated with 1 ​mL of explant media (DMEM/F12 HEPES (SH30023.01), PEST (1:100), l-Proline 0.4 ​mM, insulin, transferrin supplemented media (ITS) vitamin C 50 ​μg/mL) at 37 ​°C, 5 ​% CO_2_ for 24 ​h to equilibrate the tissue before addition of treatments. We used two previously established catabolic models [12]:

1. Interleukin-6 (IL6; 50 ​ng/mL) ​+ ​interleukin-6 receptor (sIL6R; 200 ​ng/mL), + tumor necrosis factor alpha (TNF-α; 20 ​ng/mL), hereafter referred to as IL6/TNF.
2. Oncostatin M (OSM; 10 ​ng/mL) ​+ ​TNF-α (20 ​ng/mL), hereafter referred to as OSM/TNF.

We added compounds with the potential to inhibit the catabolic processes (Fig. 1B). For all menisci (MEX5.1-MEX5.6), the molecular treatments were DEX (100 ​nM) [13], Link-N (2 ​μg/mL) and CKF (1 ​μg/mL). Thus, there were nine experimental groups in total: one control (no treatment), the two catabolic models (IL6/TNF and OSM/TNF), and the two catabolic models with each molecular treatment added (DEX, Link-N and CKF). We used one anterior and one posterior slice for MEX5.1-MEX5.3 for each treatment (A1-A9 and P1–P9; Fig. 1A), and each slice was randomized to experimental group, with meniscus part (anterior and posterior) used as block [18]. However, for MEX5.4-MEX5-6, we used single replicates (anterior part) for the molecular treatments Link-N and CKF. From the start of treatments, 80 ​% of the culture media was changed every third day. To confirm cell viability throughout the experiment, we assessed metabolic activity using the alamarBlue assay (Fig. 1C; Supplementary table 2).

### 2.3 Mass spectrometry proteomics

#### 2.3.1 Sample preparation

We pooled media from the anterior and the posterior part of the same meniscus from the same experimental group. 50 ​μL explant culture media was reduced by 10 ​mM dithiothreitol (30 ​min, at 56 ​°C), alkylated by 40 ​mM iodoacetamide (60 ​min, in dark and at room temperature) followed by quenching with an additional 10 ​mM dithiothreitol. Samples were subjected to 9 ​vol of 95 ​% ethanol with 50 ​mM NaAc to precipitate, and left overnight at −20 ​°C. The next day, samples were centrifuged, and supernatants were removed. Pellets were further washed with 70 ​% ethanol before digestion with 0.25 ​μg trypsin gold (Promega) in 0.1 ​M ammonium bicarbonate (AMBIC) pH 7.8 for 16 ​h on a shaker at 37 ​°C. After drying, samples were resuspended in 100 ​μL ammonium bicarbonate with 0.5 ​M NaCl, run through 10 ​kDa filter (PALL Life Sciences) and desalted with reversed-phase C18 cartridges (AssayMAP, Agilent Technologies) using a Bravo robot (Agilent).

#### 2.3.2 Data acquisition and database search

Discovery MS (data-dependent acquisition) was performed using a quadrupole Orbitrap benchtop mass spectrometer (Q-Exactive HFX, Thermo Scientific) with prior separation of peptides using a liquid chromatography system (NeoVanquish nanoLC, Thermo Scientific) on an analytical column (PepMap RSLC C18, 2 ​μm, 75 ​μm ​× ​25 ​cm, Thermo Scientific) coupled on-line using a nano-electrospray ion source with a column temperature at +45 ​°C (EASY-Spray, Thermo Scientific) using a flow rate of 300 ​nL/min and a 1 ​h binary gradient. We ran QC samples (100 ​ng Pierce HeLa Protein Digest Standard, 88329) approximately every tenth sample. Protein identification was performed in Proteome Discoverer 2.5 (Thermo Scientific) using two search engines in parallel: a tryptic search against the UniProt human (UP000005640 from 2021 to 01) sequence database combined with an MSPep spectral search against the NIST_human_Orbitrap_HCD_20160923 library (mass tolerance: 10 and 20 ​ppm in MS1, MS2 respectively. Other Sequest search settings were modifications: carbamidomethylation (fixed: C), oxidation (variable: M, P) missed cleavages (max 2), mass tolerance (MS1-10 ​ppm, MS2-0.02Da). Label-free protein abundance quantification was obtained by averaging peak area intensities from the top three unique peptides for each protein. The protein as well as peptide false discovery rate (FDR) was 0.01. Each meniscal experiment (i.e. experiment with one meniscus) was run and searched at a separate timepoint. The MS proteomics data presented in this paper can be found in Supplementary table 3.

### 2.4 Data and statistical analyses

Data analysis was performed in RStudio Version 2024.04.0 ​+ ​735. The *ggplot2* package was used for visualization.

#### 2.4.1 Pre-selected proteins

We developed a list of 49 proteins selected as relevant for OA (Supplementary table 4), including proteins of matrix metalloproteinases [19], cytokines [20], proteases, protease inhibitors and ECM proteins [21] and were mostly based on protein abundances in our previous work [12]. Proteins needed to have non-missing values in at least five menisci for statistical analysis; 39 of the 49 pre-selected proteins met this criterion (Supplementary table 4). We used a linear mixed effect model (*lme4* R-package) with log2 abundance of protein as outcome. Fixed effects were treatment, protein and their interaction, day (included as categorical variable), interaction between day and protein and wet weight of meniscus slices. Random intercepts were meniscus, proteins nested within each meniscus and we used random slopes for day, to allow each protein to have its own trajectory over time. The structure of the random effects was decided based on biological relevance and Bayesian Information Criterium. We used plots of residuals, random effects and fitted values to validate the model. We handled multiplicity issues through use of the mixed model for the pre-selected proteins, without further correction [22]. Given that the differences between the groups were stable over time for most proteins (Supplementary figure 1), we report all the between-group differences averaged over all days (from 3 to 18).

#### 2.4.2 Exploratory analysis

Further, in an exploratory part of the analysis, we fitted similar linear mixed effect models for all other proteins with non-missing values in at least five menisci (n ​= ​809 proteins). Here, we analyzed each protein in a separate model and, as above, results are reported averaged over days. As this part of analysis was exploratory, we did not use corrections for multiplicity. To minimize the risk of identifying misleading associations, we required a protein to be affected by both catabolic models, in the same direction to conclude that the protein is affected by the catabolic models. Similarly, the treatment-induced changes in protein levels needed to achieve statistical significance across both catabolic models.

### 2.5 Complementary targeted analyses

#### 2.5.1 GAG release

The release of GAGs was analyzed with a 1,9-dimethylmethylene blue (DMMB) assay, as previously described in Ref. [12]. To estimate differences of GAG release between treatments (Supplementary table 5), we used a linear mixed effect model. GAG release (μg/mL) was used as outcome variable and fixed effects were treatment, day, wet weight and slice (anterior or posterior position). Further, we corrected for baseline GAG release at day 0 (before addition of any treatment). Random effects were slice nested within meniscus. No major differences in the estimates were observed over day 3 until day 18 and therefore, differences in GAG release are presented as averaged over time.

#### 2.5.2 Western blot

We performed a targeted analysis of COMP fragment release using an antibody against the neoepitope QQS^77^ [23] on a subset of treatments: control, OSM/TNF and OSM/TNF ​+ ​DEX; at day 12, based on that we identified most fragments at this specific day in our previous work [12]. This decision was made as a trade-off to allow for the inclusion of more biological replicates on the same gel rather than more experimental groups. Five menisci were randomly selected for analysis. Media from the same meniscus and same experimental group were pooled (1:1) to a final volume of 9 ​μL. 3 ​μL of SDS Sample Buffer (Thermo Fisher Scientific, 1981103) was added, and samples were incubated for 10 ​min at 70 ​°C. Following, samples were run on a NuPAGE 4–12 ​% Bis-Tris Gel in MOPS buffer (Thermo Fisher Scientific, 2711057) at 200 ​V for 50 ​min. In a buffer (Thermo Fisher Scientific, 2461350) with 10 ​% methanol, proteins were transferred to a polyvinylidene difluoride (PVDF) membrane at 25 ​V for 90 ​min. The membrane was blocked with 3 ​% Bovine Serum Albumin (BSA) in 10 ​mM Tris, 0.9 ​% NaCl and 0.2 ​% Tween (pH ​= ​7.4) buffer (BSA ​+ ​buffer) for 1 ​h in room temperature. Primary antibody (in BSA ​+ ​buffer at 1:1000) incubation was carried out overnight at 4 ​°C. The next day, the membrane was washed with buffer before the secondary antibody (Donkey anti-rabbit; Jackson 711-035-152) was added (BSA ​+ ​buffer at 1:10,000) and incubated for 1 ​h at room temperature. Finally, the membrane was washed 2 ​× ​10 ​min in buffer and 1 ​× ​10 ​min in buffer without Tween, before it was subjected to a chemiluminescent substrate (SuperSignal West Dura kit) for 1 ​min and imaged using Bio-Rad, ChemiDoc MP Imaging System.

## 3 Results

### 3.1 Cell viability

All meniscus explant cultures had a higher reduction of alamarBlue compared to negative control, indicating that cells were healthy and viable throughout the whole experiment (Supplementary figure 2).

### 3.2 Identification and quantification in proteomics data

Number of identified proteins per menisci (at any day) were 988, 950, 1177, 1185, 1157 and 1875 for MEX5.1, MEX5.2, MEX5.3, MEX5.4, MEX5.5 and MEX5.6, respectively. Of these, a total of 661 proteins were shared among all six menisci. Missing values per sample ranged between 4.6 and 34.7 ​% (Supplementary table 6).

### 3.3 Differential abundance of proteins

#### 3.3.1 Pre-selected proteins

Two proteins were totally missing in the data among pre-selected proteins: CCL8 and CXCL6 (Supplementary table 7). The other eight proteins that were filtered out before statistical analysis were totally missing in MEX5.3 and were generally stable over time within the other individual menisci (Supplementary figure 3). The same can be observed for the rest of proteins (Supplementary figure 4).

**Fig. 2:**
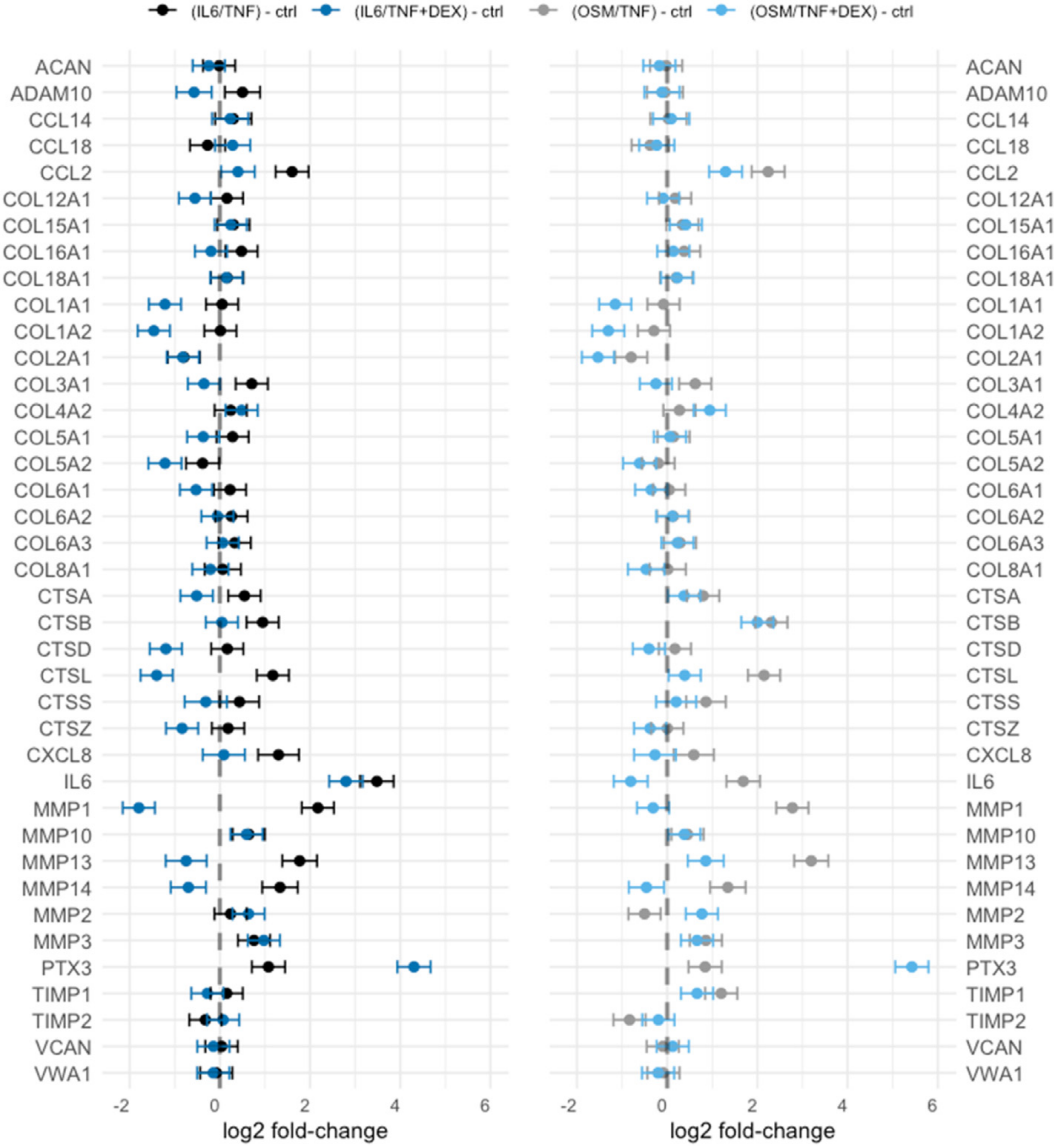
Pre-selected proteins: estimates from linear mixed effect model. Estimates are presented as log2 fold-changes contrasted between IL6/TNF vs. ctrl (black), OSM/TNF vs. ctrl (grey), IL6-TNF ​+ ​DEX vs. ctrl (dark blue) and OSM/TNF ​+ ​DEX vs. ctrl (light blue). Bars represent 95 ​% CIs. If estimates lie on the vertical axis of x ​= ​0, there is no difference to control. If an estimate lies above the vertical line (x ​> ​0), the specific protein is less abundant in control. On the other hand, if an estimate lies below the vertical line (x ​< ​0), the specific protein is more abundant in control. Further, if the estimate for a catabolic model has a higher value than the estimate for the model with addition of DEX, the specific protein is more abundant in the catabolic model. This can be observed in for example MMP14, for both catabolic models. Opposite, if the estimate for a catabolic model has a lower value than the estimate for the model with addition of DEX, the specific protein is less abundant in the catabolic model. This is the case for PTX3 for both catabolic models. IL6/TNF: interleukin-6 and TNF; OSM/TNF: oncostatin-M and TNF; DEX: dexamethasone.

We have confirmed a profound effect of the two catabolic models in menisci on the protein abundance release of important OA-related proteins, such as matrix metalloproteinases [19] and cathepsins [24] (Fig. 2). There is a larger effect (increased release) when using the OSM/TNF model on proteins CTSB, CTSL, MMP1, MMP13 and TIMP1 (Supplementary figure 5). On the other hand, the IL6/TNF model showed a higher release of ADAM10, CXCL8, MMP2 and TIMP2 (Supplementary figure 5).

No notable treatment effects with the addition of CKF or Link-N could be observed (Supplementary figure 6). On the other hand, the addition of DEX did indeed alter the protein abundance as compared to the catabolic models (Fig. 2). These were for example OA-related proteins: MMPs (MMP1, MMP13, MMP14), cathepsins (e.g. CTSD and CTSL), and the chemokine CCL2, but also less release of some collagens (especially COL1A1 and COL1A2 for both catabolic models). This indicates that the addition of DEX, to both catabolic models, reduces catabolic activity. Notably, PTX3 is considerably more abundant when DEX is added.

#### 3.3.2 Exploratory analysis

**Fig. 3:**
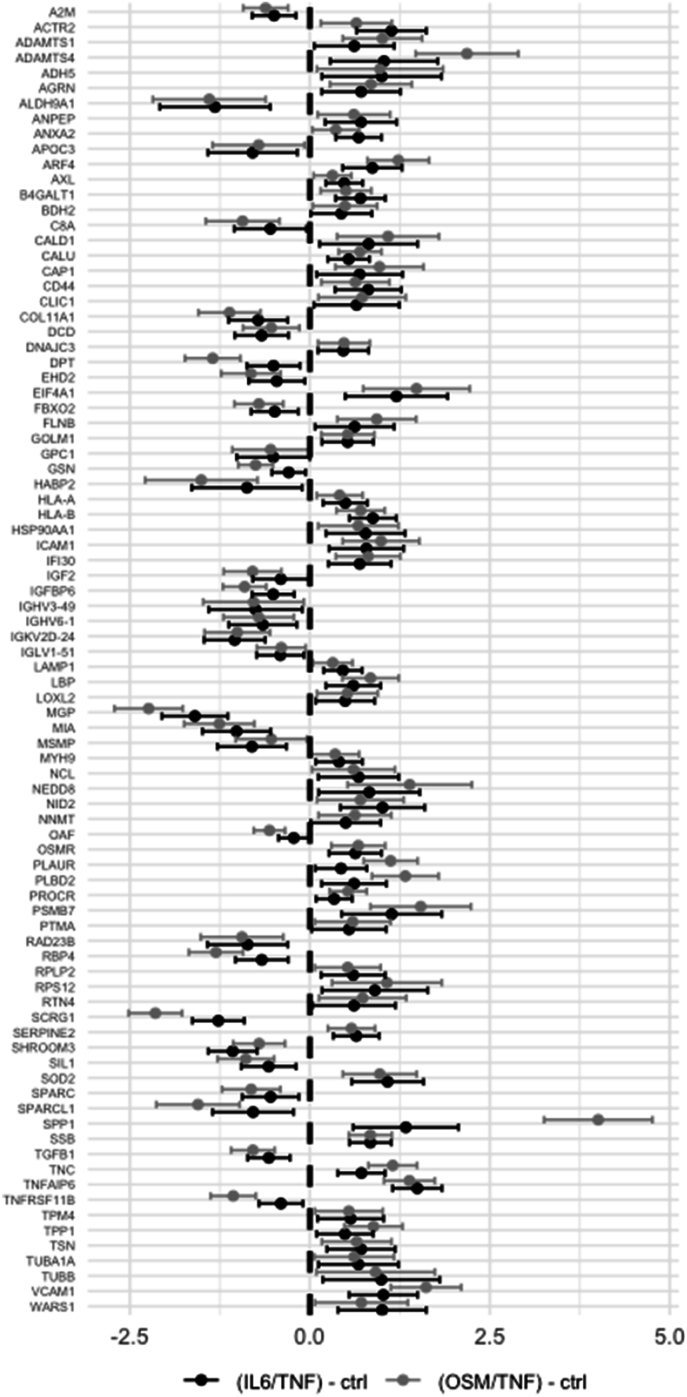
Exploratory analysis: estimates from linear mixed effect models of the 86 proteins that was found be significantly affected by both catabolic models, in the same direction. Estimates are presented as log2 fold-changes contrasted between IL6/TNF vs. ctrl (black) and OSM/TNF vs. ctrl (grey). Bars represent 95 ​% CIs. IL6/TNF: interleukin-6 and TNF; OSM/TNF: oncostatin-M and TNF.

**Fig. 4:**
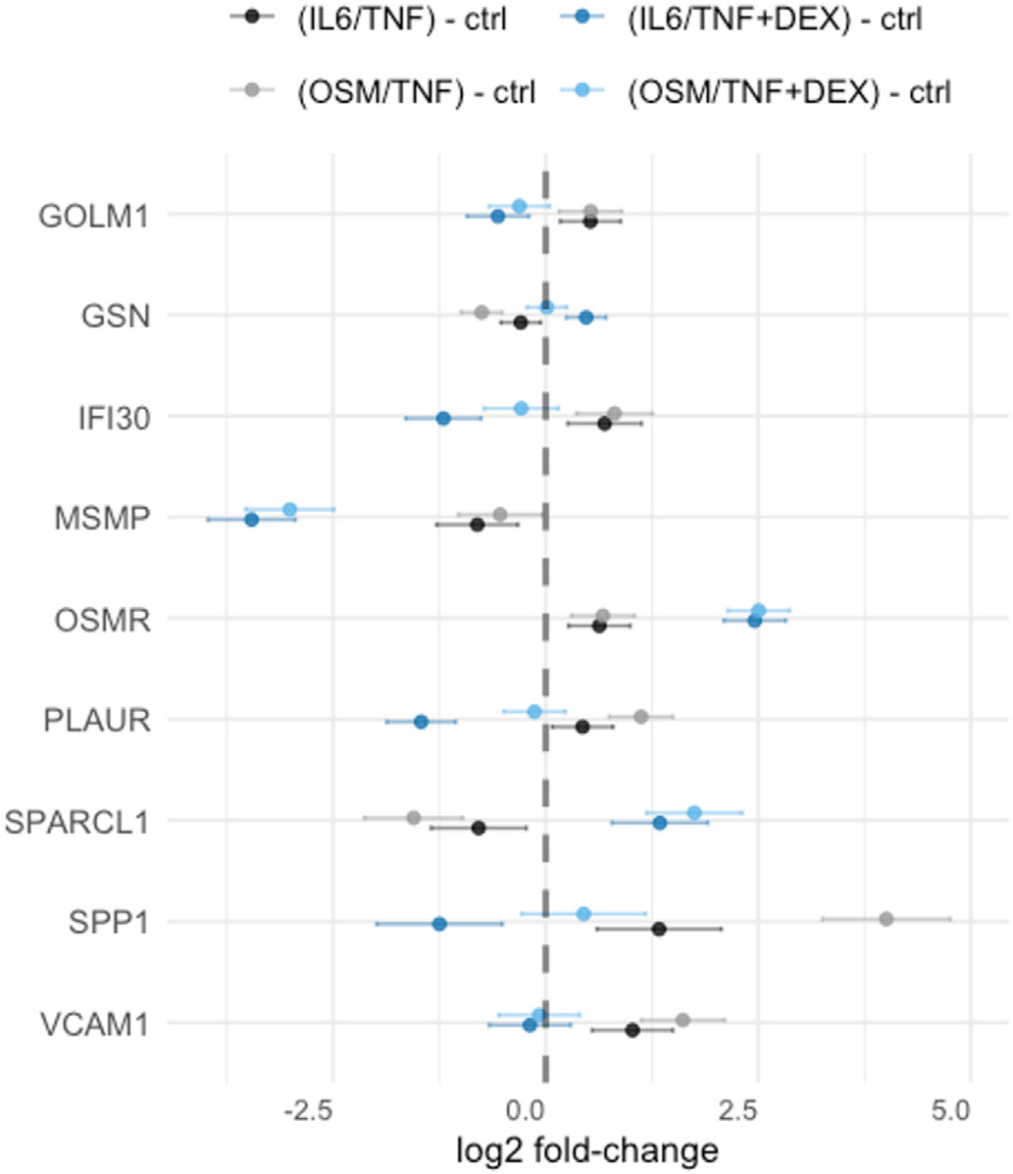
Exploratory analysis: estimates from linear mixed effect models of the proteins that were found be significantly affected by the addition of DEX in both catabolic models, in the same direction. Estimates are presented as log2 fold-changes contrasted IL6/TNF vs. ctrl (black), OSM/TNF vs. ctrl (grey), IL6-TNF ​+ ​DEX vs. ctrl (dark blue) and OSM/TNF ​+ ​DEX vs. ctrl (light blue). Bars represent 95 ​% CIs. IL6/TNF: interleukin-6 and TNF; OSM/TNF: oncostatin-M and TNF; DEX: dexamethasone.

A total of 809 proteins were quantified in at least five menisci (Supplementary table 8), excluding the pre-selected proteins. Among these, 86 proteins were statistically significantly affected by both catabolic models, in the same direction (Fig. 3). Note, SPP1 fold change is three times higher in OSM/TNF compared to IL6/TNF. Link-N and CKF treatments had little (OSM/TNF) or no (IL6/TNF) impact on the protein abundance among these 86 proteins (Supplementary figure 7), while the addition of DEX was the molecular treatment with the most substantial impact (Fig. 4). The three proteins with the largest fold-change (log2 FC) are SPP1, SPARCL1 and MSMP for both catabolic models (Fig. 4).

### 3.4 Complementary targeted analyses

#### 3.4.1 GAG release

There was generally little effect of GAG release with any of the molecular treatments in comparison to the corresponding catabolic model (Supplementary figure 8). However, GAG release is lowered when DEX is added to the catabolic model OSM/TNF, log2 FC -4.87 (OSM/TNF ​+ ​DEX vs. OSM/TNF; 95 ​% CI -7.76 to −1.98; Supplementary table 9).

#### 3.4.2 COMP fragments release

Western blot analysis of COMP neoepitope QQS^77^ showed an increase of released fragments in the catabolic model OSM/TNF at day 12 (Supplementary figure 9). Conversely, the presence of DEX appears to reduce this release in most menisci, supporting previous findings that DEX is effective in mitigating the catabolic effect.

## 4 Discussion

OA is characterized by the degradation of the ECM within joint tissues. This process is driven partly by the inflammatory system, where TNF and IL-1β have been proposed as the main cytokines to target [11]. We used IL-1β treatments in our previous work [12], but built upon the more promising OSM/TNF and IL6/TNF models in this study. We have confirmed a strong effect of the catabolic models on pre-selected proteins measured in media from meniscus cultures. Levels of cathepsins B and S were increased in the two catabolic models compared to untreated controls (Fig. 2). Others have reported that these proteins were elevated in cytokine treated chondrocytes, and cathepsin B especially was associated with OA severity in synovial fluid [24]. SPP1 (osteopontin) levels were higher in OSM/TNF compared to IL6/TNF (Fig. 3), which was not observed in our previous work where SPP1 levels were lower in catabolic models than controls [12]. The previous study used a single meniscus, while the current study's six biological replicates enhance reliability. Additionally, now we saved 20 ​% of the culture media during exchanges to retain important secreted molecules, which may explain the differences.

We investigated three molecules as potential treatments to prevent the cytokine-induced meniscal degradation processes. We found no treatment effect when using Link-N or CFK, but we found consistent strong anti-catabolic effect when menisci were treated with DEX (Fig. 2; Fig. 4). The effect of DEX was confirmed by the measurement of GAG release in the OSM/TNF catabolic model. To further validate the effect of DEX, we used an antibody against the neoepitope QQS^77^ of COMP fragments [23] and generally observed a decrease in the release of fragments into the culture media. Further, a large increase of PTX3 is observed when DEX was used as a treatment compound. Increase of PTX3 has also been observed in human aortic smooth muscle cells [25] and human fibroblast cells [26] with DEX treatment. Interestingly, an enhanced production of the protein was observed in human lung fibroblast cells when DEX was added together with TNF-α or IL-1β [26]. In a knockout OA mouse model, the absence of the PTX3 gene was found to reduce the severity of cartilage degeneration [27], suggesting that the effect of increased PTX3 expression by DEX could be unfavorable.

A peptide from the Link protein (DHLSDNYTLDHDRAIH) has been reported to mitigate catabolic processes in human OA cartilage explants treated with cytokines by preventing GAG loss, as well as increasing the gene expression of aggrecan and decreasing the expression of proteolytic enzymes in chondrocytes [15]. While we used the peptide DHLSDNYT, referred to as Link-N, the difference in length could explain the lack of response in our meniscus explants. Another explanation might be that neither of the peptides are effective in mitigating catabolic responses in meniscus. While the inner zone of the meniscus is similar to cartilage, primarily composed of collagen type II and chondrocyte cells, the outer zone has a distinct composition. The outer zone is vascularized and contains fibroblast-like cells and collagen type I as the main cellular and structural components [1]. Further, cartilage consists of about 10–15 ​% of negatively charged proteoglycans [28], whereas they are much less common in the meniscus with only 1–2 ​% of its dry weight [29]. For the major proteoglycan aggrecan there is a 10-fold higher level in articular cartilage compared to the meniscus measured by quantitative proteomics [30]. The Link-N peptide is part of the Link protein, which helps stabilize the proteoglycan aggregates in cartilage. The lack of effect in meniscus could be due to the large variation in proteoglycan present in the two tissues. It is also possible that the menisci in this study, starting from a relatively healthy state, is non-comparable with OA cartilage, even for the cartilage-like zone. CKF, a peptide from the protein chondroadherin, appears naturally as a cleavage product in cartilage, but it is unknown if the fragment itself possesses any role in cell regulation within the tissue [16]. Furthermore, the chondroadherin level in the meniscus is markedly lower than in articular cartilage [30]. Taken together, this may explain the lack of response in the current study involving meniscal tissue.

In the exploratory analysis, the same three proteins (SPP1, SPARCL1 and MSMP) had the highest absolute log2 FC for treatment of DEX in comparison to the catabolic models (Fig. 4). SSP1 is both a member of the ECM and a soluble cytokine [31]. Levels of this protein were found to be lower in synovial fluid at time of surgery compared to an initial physician visit after an anterior cruciate ligament tear [32], and in a post-traumatic OA model of human ankle cartilage compared to untreated controls [33], suggesting its role in acute inflammation. SSP1 was increased in both catabolic models (Fig. 3) and the addition of DEX decreased the release to the media (Fig. 4). MSMP (prostate-associated microseminoprotein) is a ligand for C–C chemokine receptor type 2 (CCR2) and regulates the migration of certain immune cells [34]. MSMP has been reported to be downregulated on the RNA level comparing OA to non-OA cartilage [35]. In our work, MSMP was decreased in the media for both catabolic models, and the addition of DEX further decreased the levels. SPARCL1 (secreted protein acidic rich in cysteine-like 1) was increased when DEX was added, and has previously been identified in synovial fluid from OA patients [36]. This protein has been suggested to have tissue dependent functions and it has been observed to be increased in OA patients contrasting damaged cartilage compartments compared to undamaged ones, and led to ECM degradation [37]. SPARCL1 has also been found to be upregulated 5-fold on the RNA level in the cartilage-like white-white zone in human OA menisci as compared to non-OA controls [38].

DEX has been investigated as a treatment in post-traumatic OA porcine explant models [39] and the authors reported that cytokines and aggrecanases were downregulated 8 ​h after injury when DEX was used as treatment, compared to uninjured controls. Another study using Western blot analysis showed that IL-1β-treated meniscus explants revealed a reduction in COX-2 (involved in inflammation) levels following DEX treatment [6]. The use of DEX as a treatment for OA is debated due to the potential cytotoxic side effects [13]. One study found that treating human meniscal cells over two weeks with DEX (at 10x higher levels than in our study) led to an increase in both cell autophagy and apoptosis [40]. We could not see any indication that DEX specifically affected cell viability differently from other treatments (Supplementary figure 2). A major difference is that we kept the meniscal cells in their native ECM environment, while the authors in Ref. [40] used isolated cells for their culture experiments. A potential approach to mitigate the potential cytotoxic side effects of DEX involves using targeted and controlled drug delivery carriers with release of the therapeutic drug over a time interval or in response to *in vivo* conditions [41,42]. DEX with a drug delivery system was tested in bovine cartilage explants, where one system improved delivery 8-fold compared to soluble DEX alone [43], and another controlled DEX release with specific linkers [44]. We believe DEX is a potential candidate drug to be considered when there is major inflammation present (or to be expected) e.g. post-injury to mitigate the initial inflammatory and catabolic responses, thereby potentially reducing the risk of developing post-traumatic OA [13]. However, clinical trials are needed before any treatment recommendations can be made. Additionally, steroids like DEX are sometimes used in the management of other forms of arthritis, with a strong inflammatory component such as reactive and rheumatoid arthritis.

One advantage of our model is the use of healthy human meniscus tissue, allowing us to induce catabolic effects starting from a likely healthy baseline. While OA develops over a long period of time, our experiments are conducted over approximately three weeks. We induce OA-alike onset using harsh cytokine treatments to induce inflammation during a relatively short time period and thus mimic early stages of the disease development. We use concentrations that are higher than those observed in OA [45] and knee-injured patients [46], which is a necessary trade-off to have feasible experiment duration. Our sample size is small, but the matched design combined with block randomization of meniscus slices resulted in narrow confidence intervals. Further, in the exploratory analysis, we required a protein to be affected by both catabolic models, in the same direction, to minimize the risk of false positives. Additionally, we used slices from one meniscus as their own control, removing confounding effects and thus, improving the internal validity of the results. An important limitation with our study design is that we cannot conclude if the analyzed proteins are pre-existing tissue proteins within the explant ECM, or if a protein is newly synthesized. This is particularly relevant for the GAG analysis, as proteoglycan levels have been reported to increase in the meniscus with the progression of OA [17,47]. Further, we do not include any biomechanical aspect in this work, which is a crucial feature in cell behavior within joint tissues [48], which could be both an interesting and potentially important aspect to include in future studies. Other future aspects include long-term treatment effects and eventually whole joint effects.

In conclusion, our work demonstrates a catabolic response of IL6/TNF and OSM/TNF-treated human menisci. We tested three different molecular treatments: a Link-N peptide (Link-N), the C-terminal sequence of chondroadherin (CKF) and dexamethasone (DEX). We found no treatment effects with Link-N or CKF. However, DEX was effective in mitigating the catabolic response. DEX as a potential disease-modifying treatment in the degenerative meniscus needs to be further explored.

## Author contributions

All authors were involved in the design and interpretation of the data. The main contributors to the study design were, however, PÖ, KL, AT and ME. KL conducted the experimental work and PÖ ran the samples on the MS instrument and searched the raw data. Statistical analyses were performed by AS and AT. Data visualization and manuscript drafting were performed by AS. All authors critically revised the manuscript and gave final approval of the manuscript for submission.

## Declaration of Generative AI in scientific writing

During the preparation of this work the author(s) used Microsoft Copilot, powered by GPT-4, to improve the quality and readability of the text by asking Copilot to present alternative versions to one or a couple of sentences at a time. After using this tool/service, the author(s) reviewed and edited the content as needed and take(s) full responsibility for the content of the publication.

## Role of the funding source

The funders did not participate in the experimental work, data analysis, manuscript preparation, or publication of this study.

## Declaration of competing interest

AT serves as associate editor for statistics for the journal Osteoarthritis and Cartilage. ME reports consultancy for Grünenthal Sweden AB and Key2Compliance AB. The other authors declare no conflicts of interest.
