# Supplementary material for: Molecular treatments to reduce catabolic effects in human meniscus explant models
Source: Osteoarthr Cartil Open. 2025 Apr 30;7(3):100618. doi: 10.1016/j.ocarto.2025.100618 (PMC12413724; doi:10.1016/j.ocarto.2025.100618)
Supplement: Multimedia component 1 [file mmc1.docx]

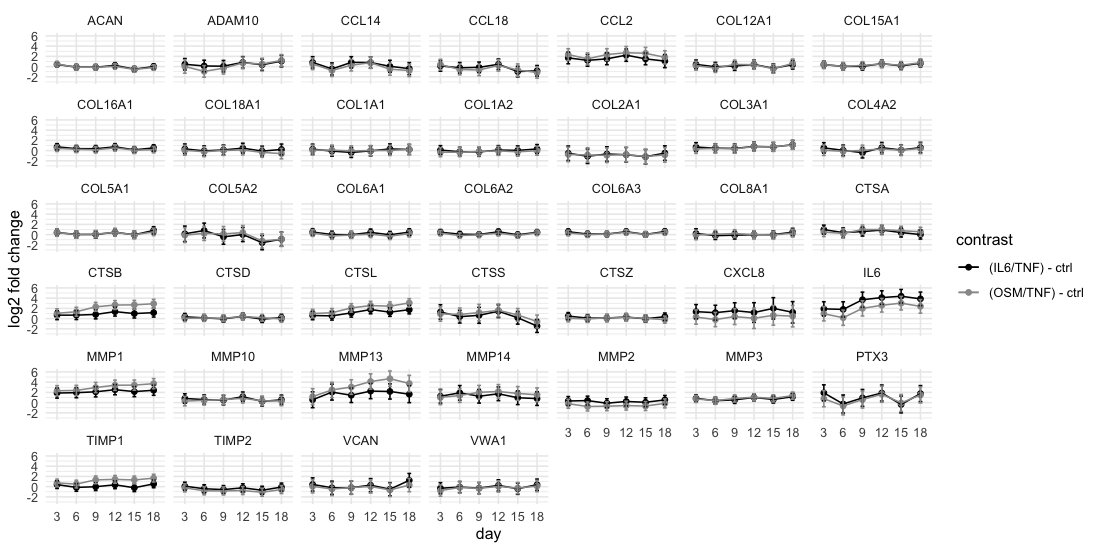


***Supplementary figure 1.*** *Pre-defined proteins: estimates from linear mixed effect model. Estimates are presented as log2 fold-changes contrasted IL6/TNF vs. ctrl (black), OSM/TNF vs. ctrl (grey) and presented per day. The between group differences are stable over time for most proteins. Bars represent 95% CIs. IL6/TNF: interleukin-6 and tumor necrosis factor alpha; OSM/TNF: oncostatin-M and tumor necrosis factor alpha.*


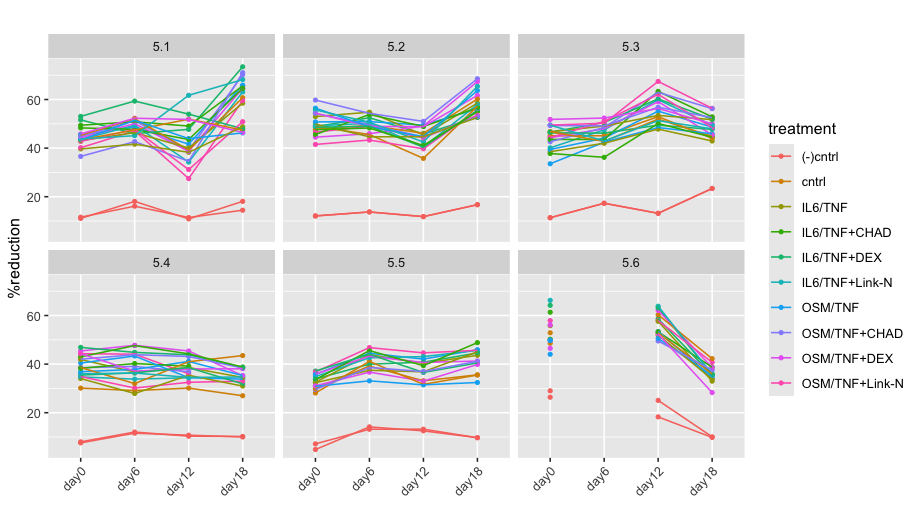
***Supplementary figure 2.*** *Reduction of alamarBlue (y-axis) throughout the whole experiment of day 0 to day 18 (x-axis). Two negative controls (no tissue) were used for MEX5.1, MEX5.4, MEX 5.5 and MEX 5.6, whereas only one was used for MEX 5.2 and MEX5.3. Note, the reduction of alamarBlue is consistently lower in negative controls compared to explant tissue cultures. Data points at day 6 for MEX 5.6 were excluded due to data acquisition at the incorrect wavelength.*

***Supplementary figure 3.*** *Pre-defined proteins: proteins measured by mass spectrometry in explant media and that were quantified in* ***less*** *than five menisci (Supplementary table 3). Explant media was collected at different time points (x-axis). Values on y-axis are log2-transformed. Note, all proteins were totally missing in meniscus Mex5.3.*


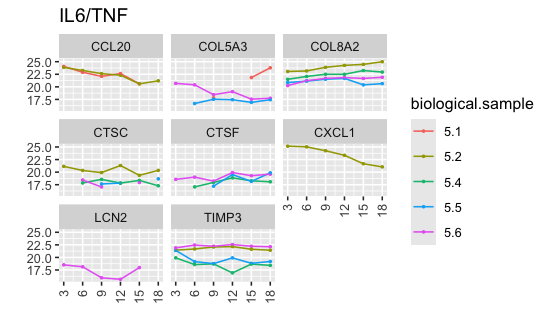

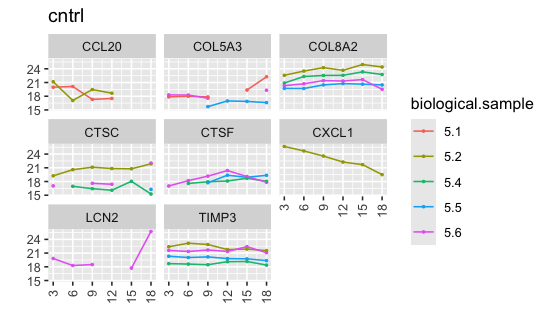

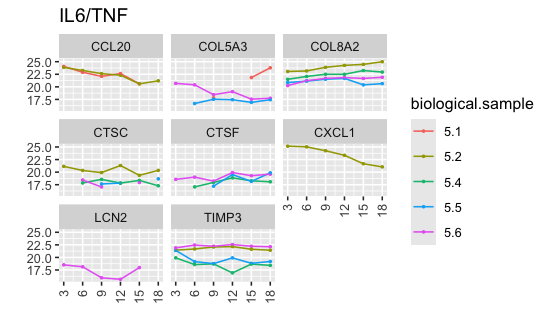

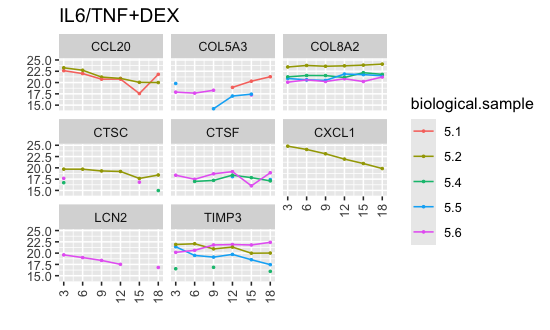

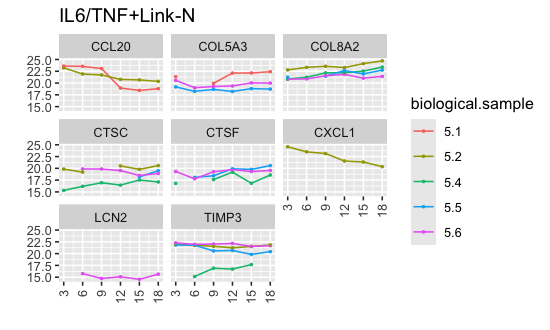

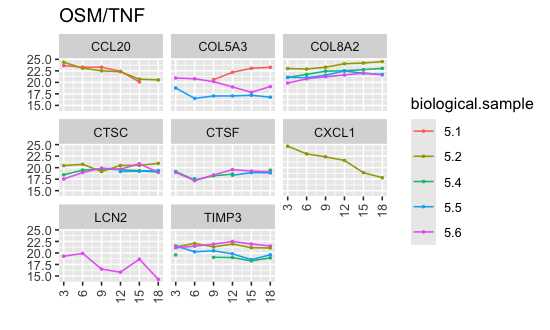

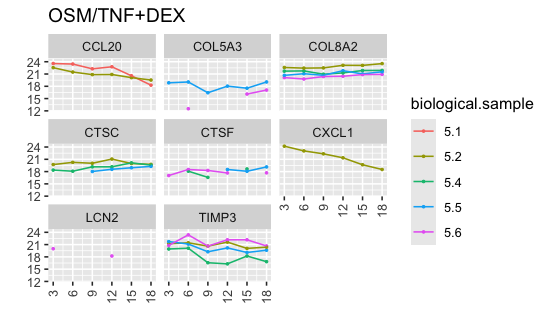

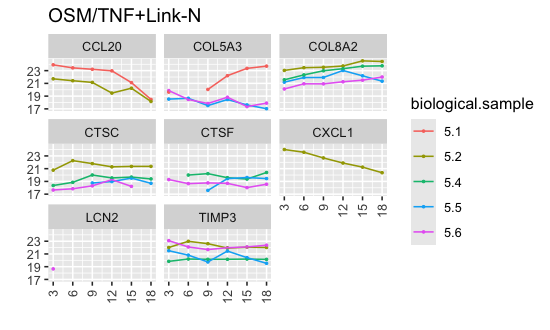

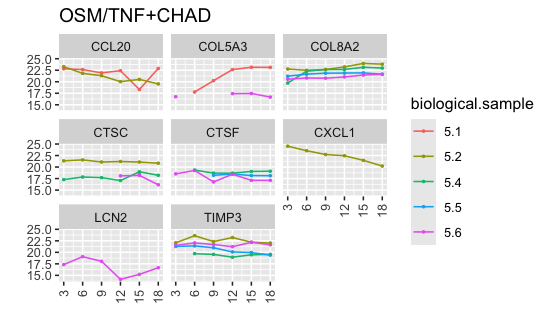

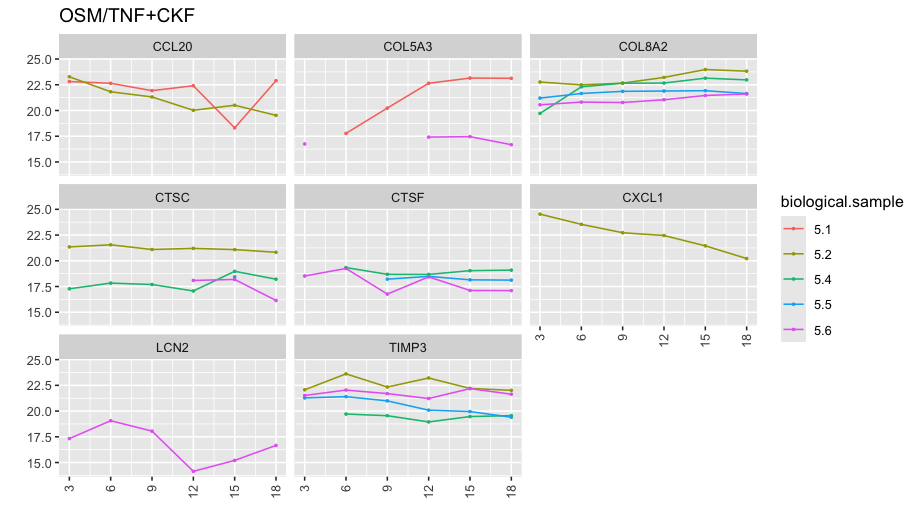

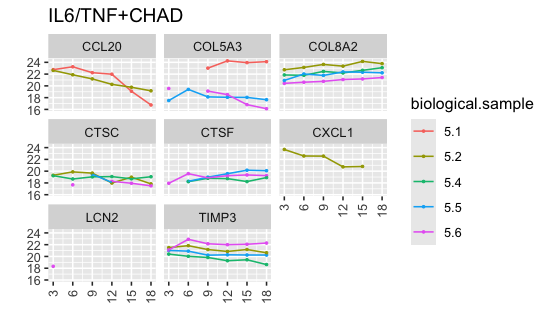

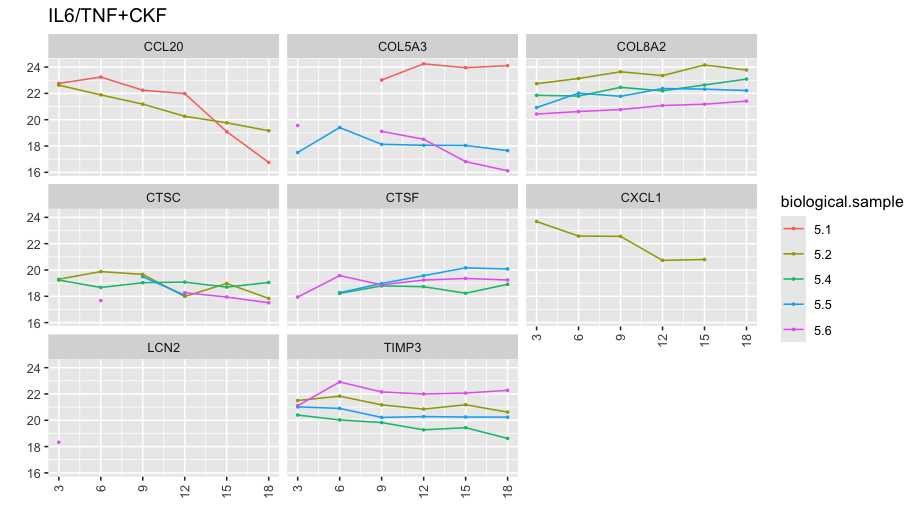

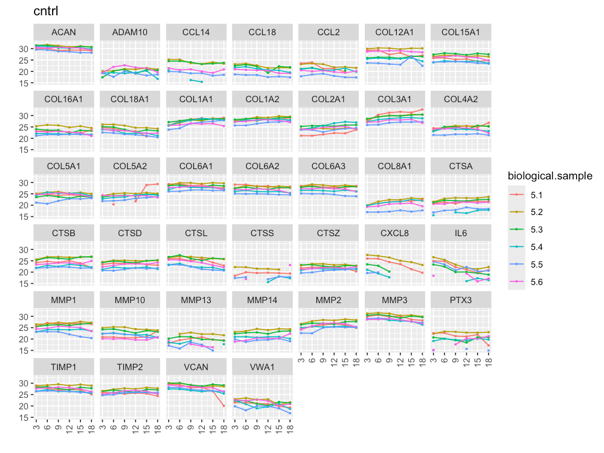

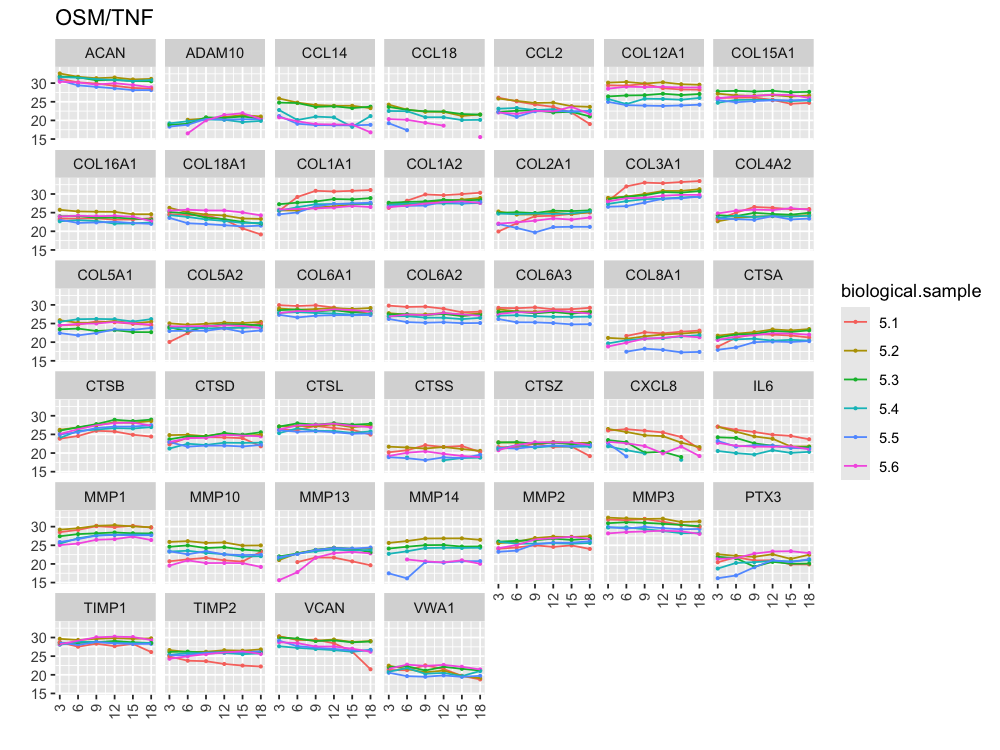

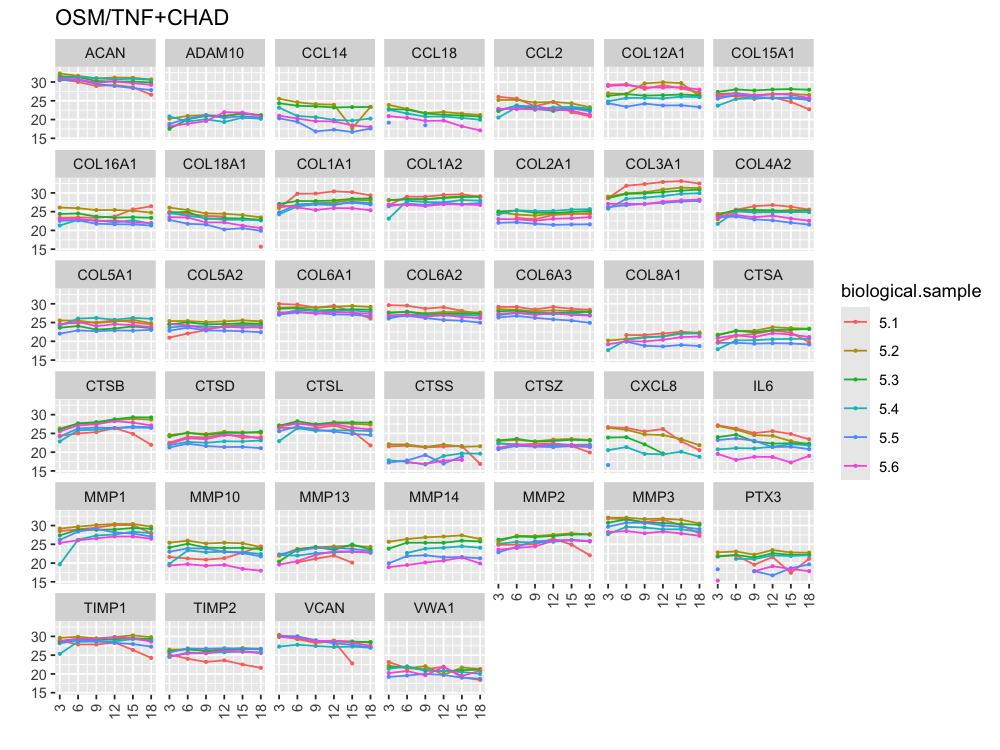

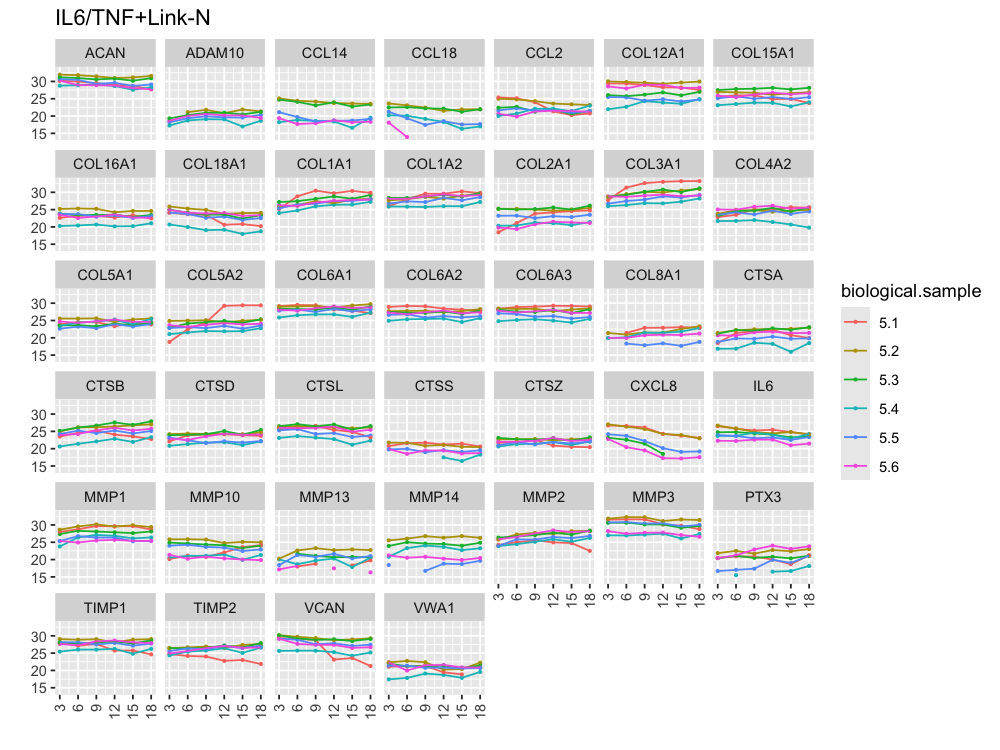

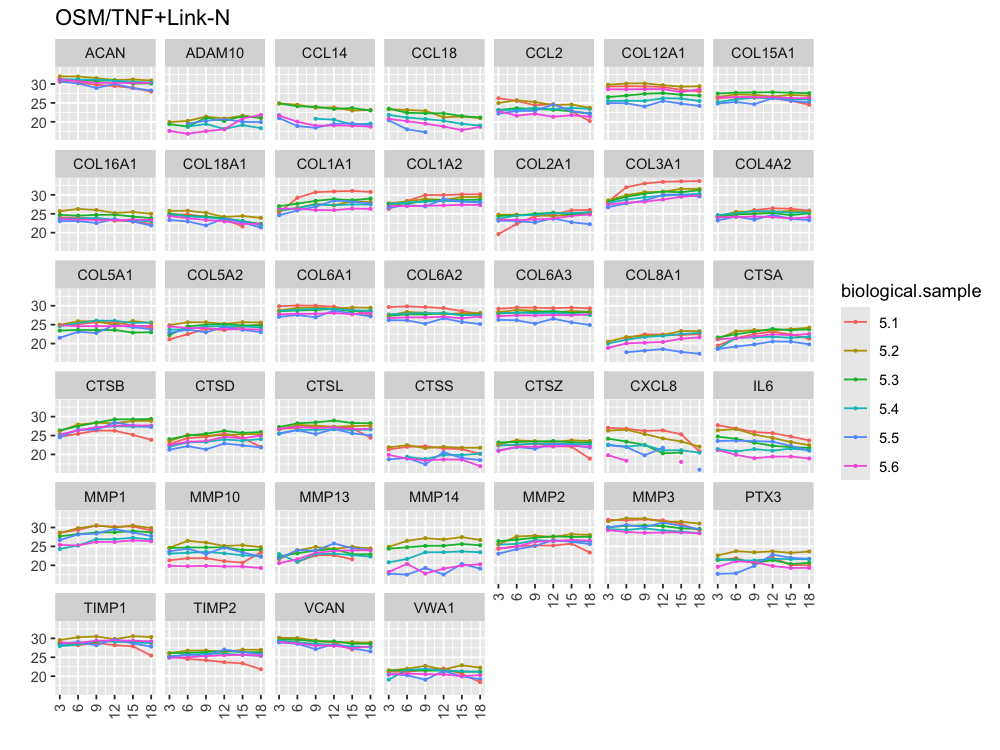

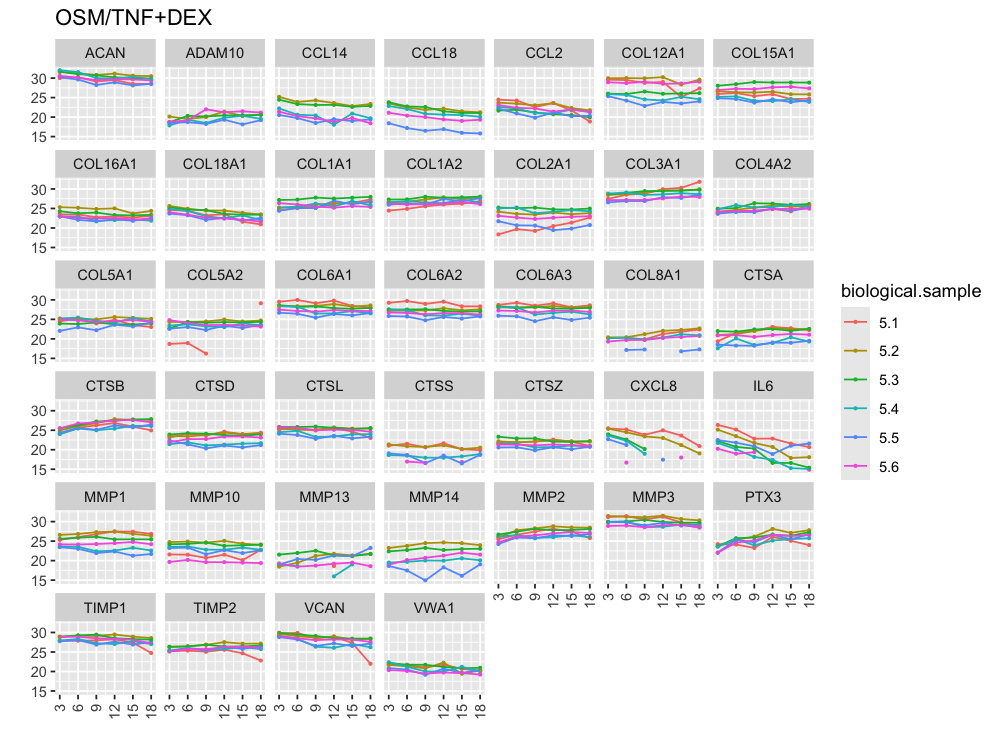

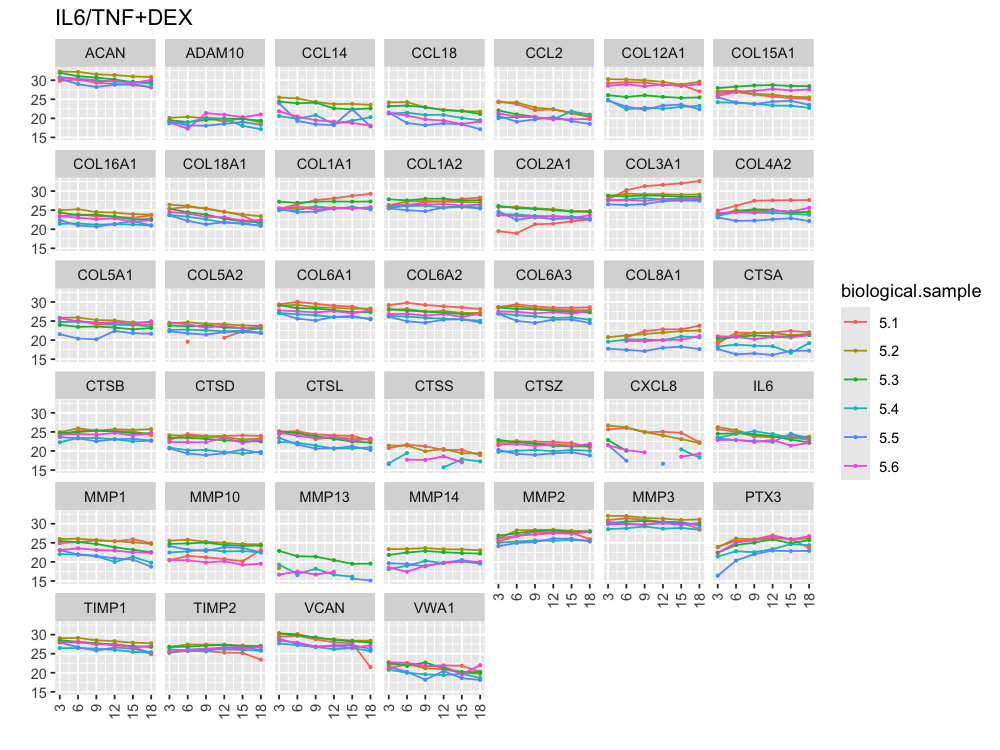

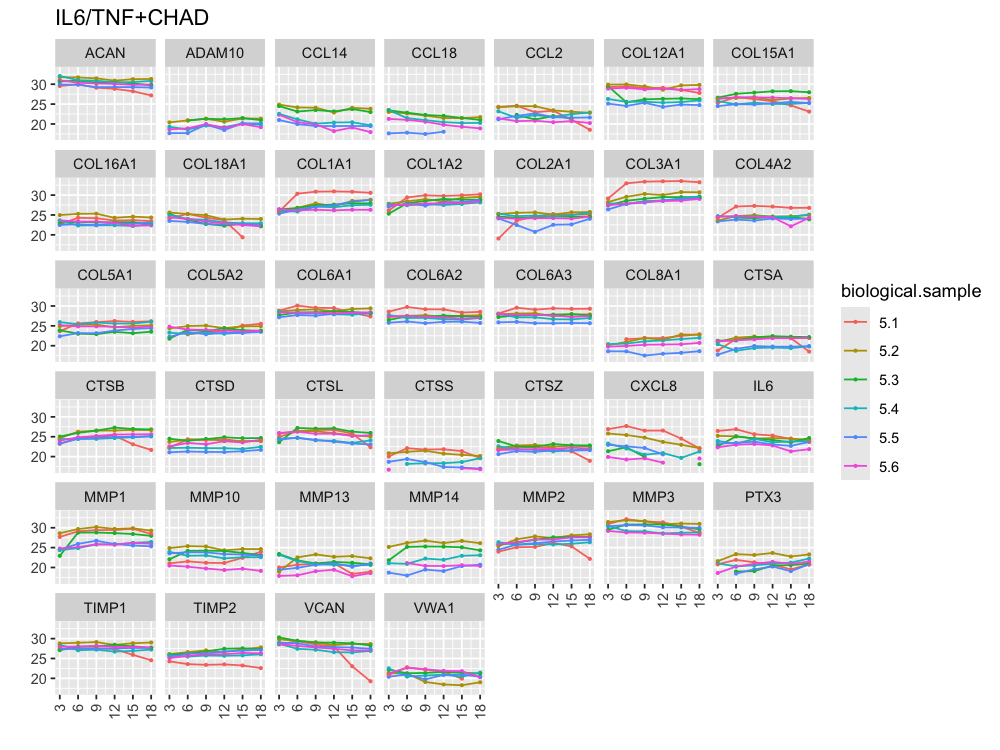


***Supplementary figure 4.*** *Pre-defined proteins: proteins measured by mass spectrometry in explant media and that were quantified in* ***more*** *than five menisci (Supplementary table 3). Explant media was collected at different time points (x-axis). Notably, the measured protein intensities were relatively stable over time within an individual meniscus, for most proteins.*


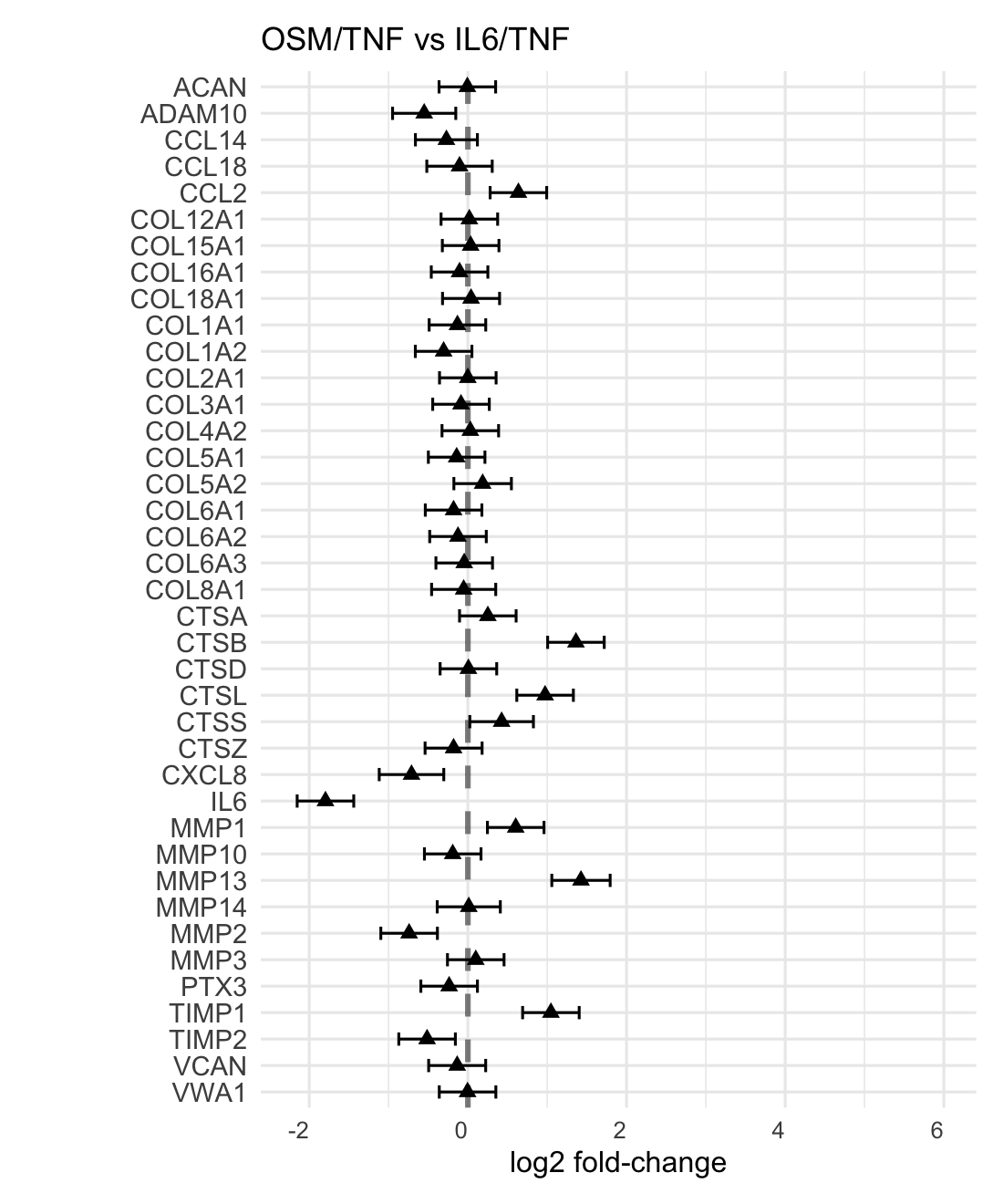


***Supplementary figure 5.*** *Pre-selected proteins: estimates from linear mixed effect models. Estimates are presented as log2 fold-changes contrasted between OSM/TNF in comparison to IL6/TNF. Bars represent 95% CIs. If estimates and confidence intervals lie on the vertical axis of x=0, there is no difference between the two catabolic models. However, an increase in release for OSM/TNF is observed for CCL2, while an increase in release for IL6/TNF is noted for ADAM10, illustrating the differences. IL6/TNF: interleukin-6 and tumor necrosis factor alpha; OSM/TNF: oncostatin-M and tumor necrosis factor alpha.*


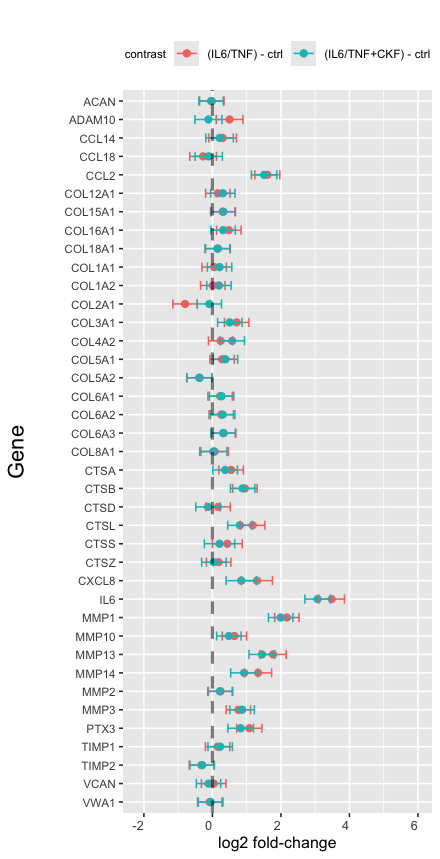

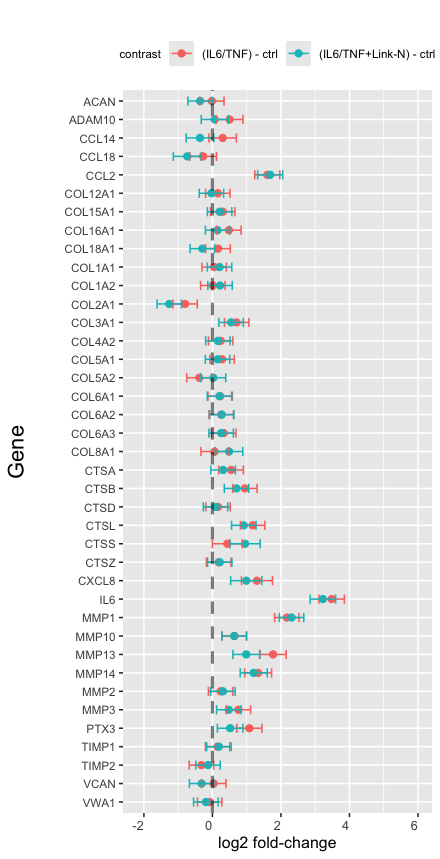

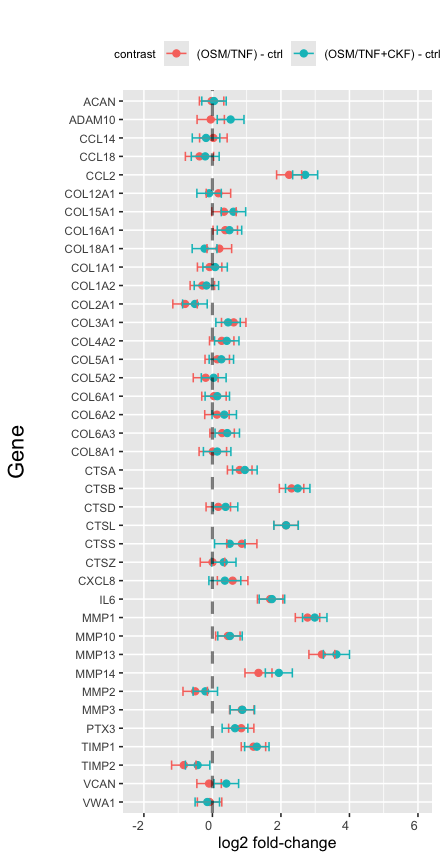

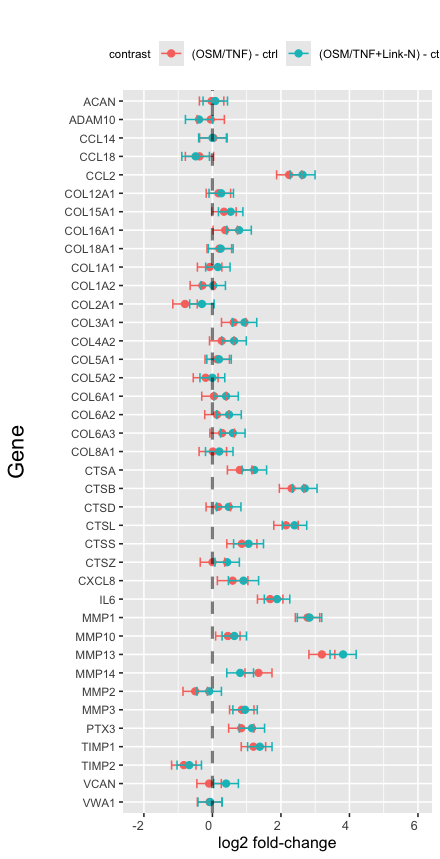


***Supplementary figure 6.*** *Pre-selected proteins: estimates from linear mixed effect models of the. Estimates are presented as log2 fold-changes contrasted between control in comparison to catabolic models (red) and addition of molecular treatment (blue). Bars represent 95% CIs. If estimates lie on the vertical axis of x=0, there is no difference to control. Here, the 95% CIs overlap between the catabolic model and the model with addition of molecular treatment for all proteins. Hence, the molecular treatment did not affect the protein abundance as compared to the corresponding catabolic model.*


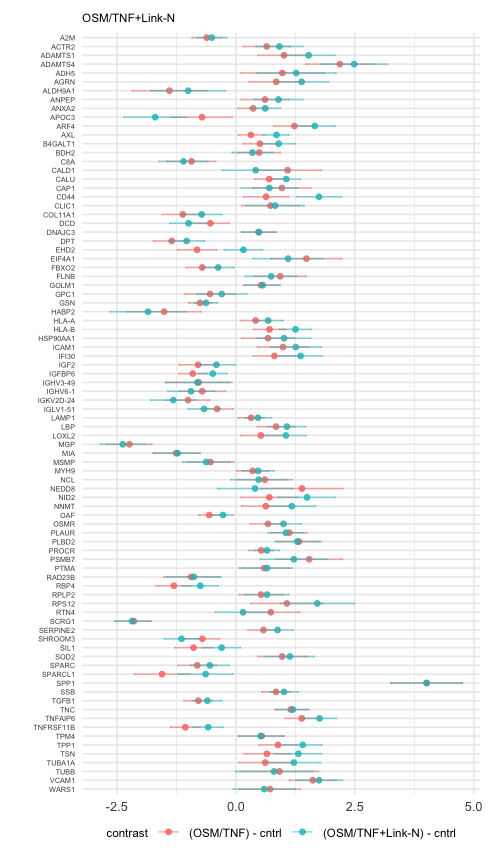

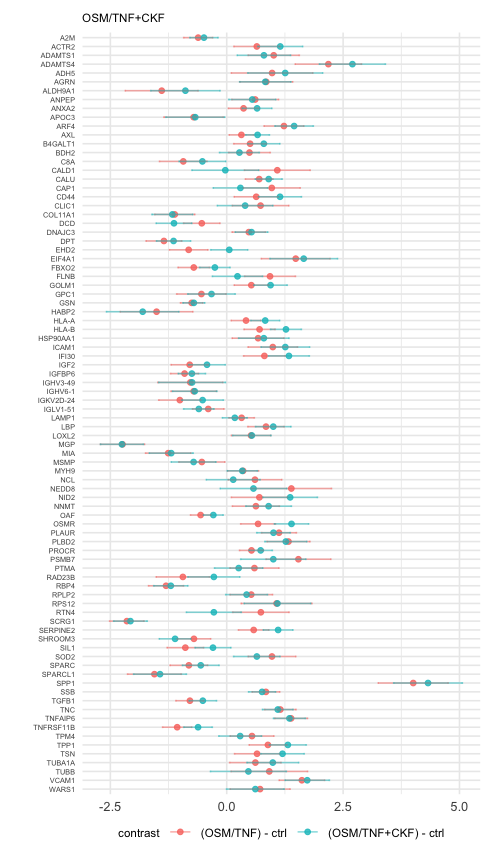

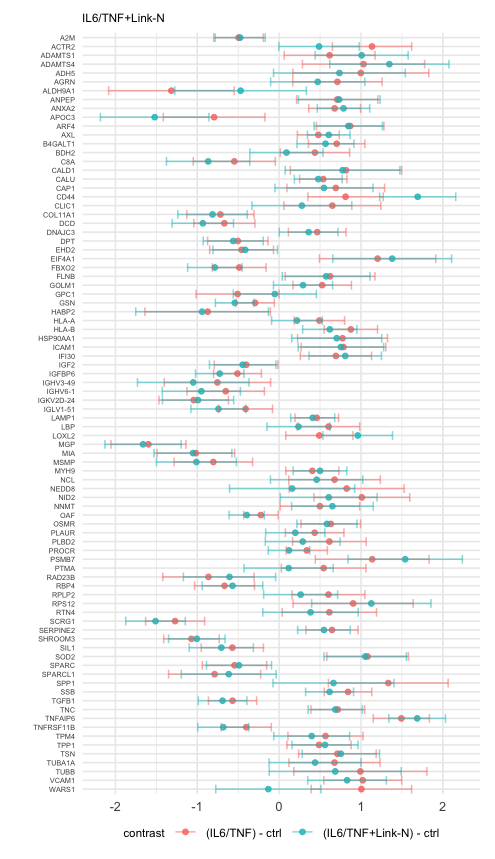

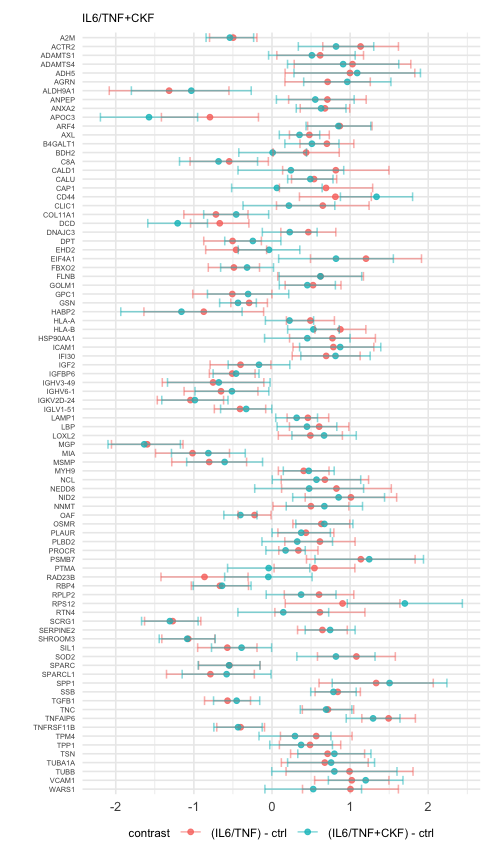


***Supplementary figure 7.*** *Exploratory analysis: estimates from linear mixed effect models of the 86 proteins that was found be significantly affected by both catabolic models, in the same direction. Estimates are presented as log2 fold-changes contrasted between 1) catabolic models vs. control (red) and catabolic model with molecular treatment vs. control (blue). Bars represent 95% CIs. For the catabolic model IL6/TNF, the 95% CIs overlap between the catabolic model and the model with addition of molecular treatment for all proteins. Hence, the molecular treatment did not affect the protein abundance as compared to the corresponding catabolic model. For the catabolic model OSM/TNF, the addition of CKF statistically significantly affected one protein (EDH2; red rectangle). The addition of Link-N to the OSM/TNF catabolic model did significantly affect three proteins (AXL, CD44 and EHD2; blue rectangles). IL6/TNF: interleukin-6 and tumor necrosis factor alpha; OSM/TNF: oncostatin-M and tumor necrosis factor alpha. CKF: peptide from chondroadherin.*


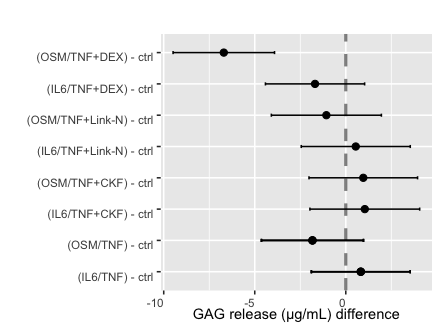


***Supplementary figure 8.*** *Glycosaminoglycan (GAG) release measured in culture media by a 1.9-dimentylmethylene blue assay*. *Difference in the average* *release of GAG (µg/mL) (y-axis) contrasting different treatments (x-axis). Bars show 95% CIs.*


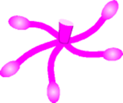

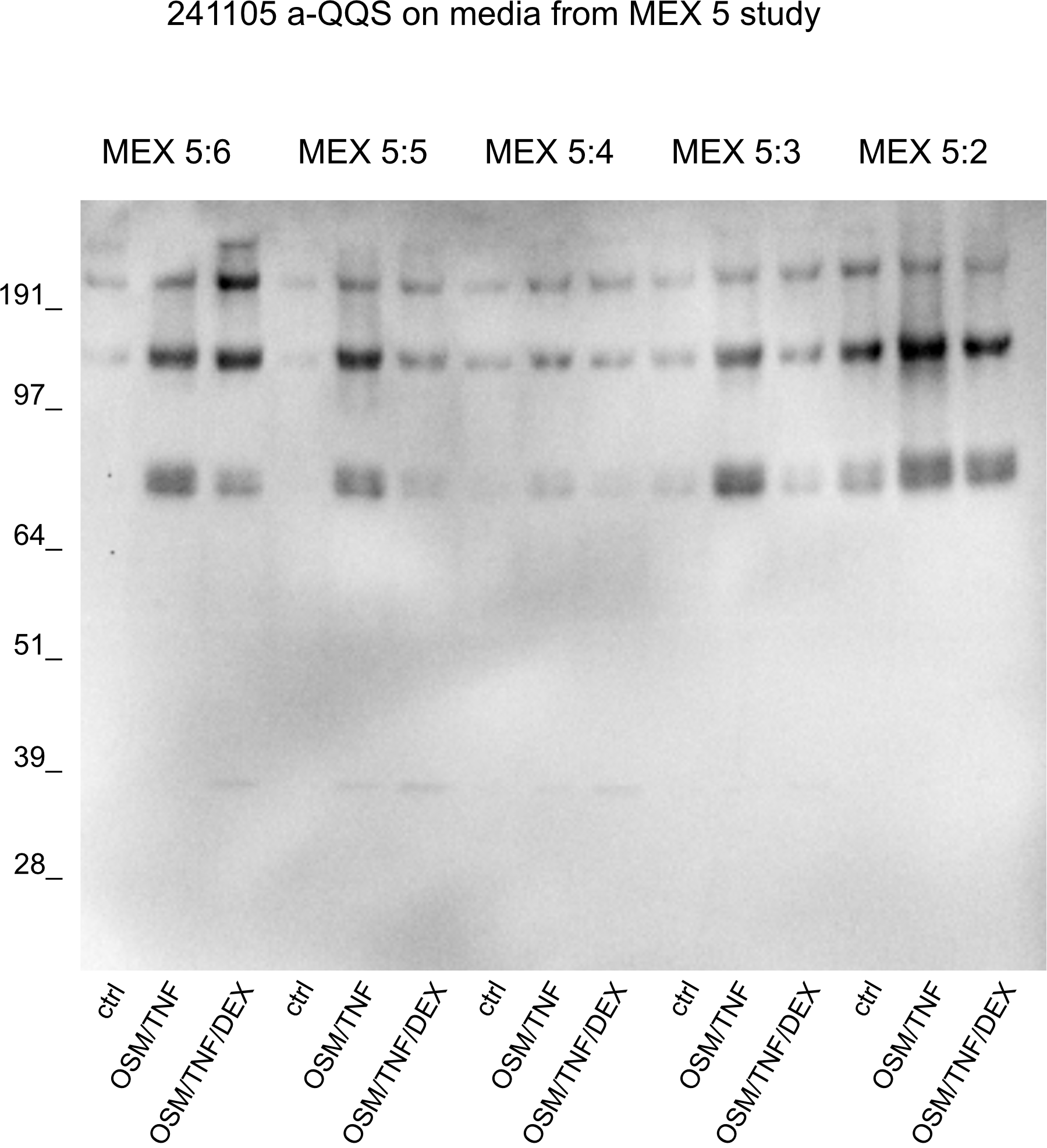

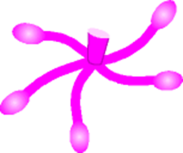

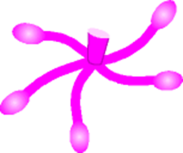

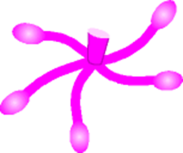


**Intact COMP**


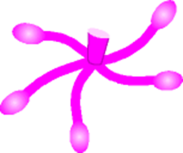


***Supplementary figure 9.*** *Western Blot of COMP fragment release (neoepitope QQS^77^) at day 12. COMP pentamer with different number of fragments cleaved can be observed. For each biological replicate (each meniscus), control show the lowest level of released COMP fragments. An increase in fragments can be observed with OSM/TNF treatment. Generally, the addition of DEX results in less amounts of released fragments. OSM/TNF: oncostatin-M and tumor necrosis factor alpha; COMP:* cartilage oligomeric matrix protein
